# Evolution of scaffold materials for periodontal therapeutics over two decades (2005–2025): A bibliometric mapping analysis

**DOI:** 10.1007/s44445-026-00235-y

**Published:** 2026-09-21

**Authors:** Yiran Lu, Xueyi Li, Ruyiren Hu, Jie Huang, Feifei Zeng, Liqi Wang, Shuo Yang, Yuting Yang, Weilian Sun

**Affiliations:** 1 The Second Affiliated Hospital Zhejiang University School of Medicine, Hangzhou, China; 2 Hangzhou Medical College, Hangzhou, China; 3 Imperial College London, London, UK; 4 The First Affiliated Hospital of Zhejiang Chinese Medical University, Hangzhou, China

**Keywords:** Periodontitis, Tissue engineering, Scaffold materials, Bibliometrics, Research trends

## Abstract

This study employed bibliometric methods to summarize the global distribution patterns, knowledge systems, and dynamic changes in hotspots of research on periodontal treatment scaffold materials over a 20-year period (2005–2025). Relevant literature related to periodontal diseases and scaffold materials was from the Web of Science Core Collection. A total of 528 eligible English-language articles were identified and analyzed using CiteSpace and VOSviewer software for visualizing and interpreting annual publication volume, international and institutional collaborations, authors, co-cited journals, and keywords. The results indicated that the number and influence of publications significantly increased from 2015, with over 60% of total publications occurring between 2020 and 2024. China and the U.S. were the top publishing countries, and international collaborative research, especially between Europe and the U.S. and between Europe, the U.S., and China, emerged as a crucial means to drive future innovation. High-frequency keywords revealed evolving themes in this field: initially focusing on basic research such as in vitro studies and stem cell differentiation, then moving towards clinical applications like bone regeneration and delivery systems, and in the last five years (post-2020), mainly centered around functional biomaterials, including hydrogels, chitosan, nanocomposites, and three-dimensional (3D)-printed scaffold materials, combined with antibacterial, immunomodulatory, and regenerative properties. The research in periodontal scaffold materials has shifted from basic in vitro experiments to clinical translational regenerative strategies. Future research will focus on developing multifunctional, personalized smart scaffold materials through continuous international and interdisciplinary collaboration and integrating them into comprehensive regenerative therapies, aiming to improve the clinical results of periodontal therapy.

**Supplementary Information:**

The online version contains supplementary material available at https://doi.org/10.1007/s44445-026-00235-y.

## Introduction

Periodontal diseases, are one of the most common chronic inflammatory conditions worldwide, are divided into gingivitis and periodontitis according to the regions of involvement(Howard et al. 2021), with significant morbidity and impact on patients’ life quality (Eke et al., 2012; Global Burden of Disease Study 2013 Collaborators 2015). The periodontal tissues consist of four constituents: periodontal ligament fibers, alveolar bone, cementum, and gingiva(Abedi et al. 2022). For detail, gingivitis, an early and reversible stage of inflammation, causes inflammation of the gums if not treated in time(Papapanou et al. 2018). And periodontitis irreversibly destroys the alveolar bone and periodontal ligament, which ultimately results in the tooth displacement and loss(Suh et al. 2019). But these damaged complex three-dimensional tissues cannot regenerate spontaneously or be fully restored by conventional clinical therapies, thus the repair of periodontal tissue represents a pivotal challenge in the treatment and research of periodontitis.

Tissue engineering, a branch of regenerative medicine, offers a promising strategy for repairing and regenerating periodontal tissue(Chen et al. 2024; Puterman et al. 2023), combining scaffold materials(Rasperini et al. 2015; Staples et al. 2020; Woo et al. 2021; Xu et al. 2021), cells(Chamila Prageeth Pandula et al., 2014; Cho et al. 2019; Wang et al. 2023), and growth factors. It is worth noting that scaffold materials play a pivotal role to create a three-dimensional biomimetic environment that induces natural healing processes and new tissue formation in the body. Periodontal tissue regeneration is achieved when scaffold materials provide both mechanical support and instructive signals that guide cell recruitment, proliferation, and differentiation(Sheehy et al. 2025). Although various studies have reported the development of materials in periodontal tissue engineering, these studies remain broad and fragmented, focusing on diverse materials, manufacturing technologies, etc.(Chen et al. 2024; Yu Wang et al. 2025a, b). Few systematic evolutions have summarized the current status and trends of research, clouding the research status, evolution, and future trend.

Bibliometrics is a method that systematically analyzes and quantifies research literature. Web of Science (WoS) is the most widely used database for this kind of work, simply because it covers more publications compared to other databases, which renders it a robust source for bibliometric analysis(Dong et al. 2019; Perazzo et al. 2019). For instance, CiteSpace and VOSviewer have been employed to assess research patterns and predict future trends in scientific publications (Agarwal et al. 2016; van Eck and Waltman 2010).

Therefore, we applied bibliometric analysis to systematically observe and visualize the evolution of research on scaffold materials in periodontal treatment. We used CiteSpace and VOSviewer to examine the literature from the WoS Core Collection (2005–2025). Our analysis looked at annual publication volume, country and institutional distribution, author and journal networks, disciplinary categories, and keyword frequencies. From the bibliometric analysis (Sect.  3), we identified the key research hotspots and traced the development trend, the research of scaffold materials for periodontal treatment were also identified, which provide new research directions for future studies aimed at informing clinical practice and guiding scaffold-based therapies.

Compared with previous bibliometric studies on periodontal regeneration, this work innovatively narrows the research scope to exclusively focus on periodontal scaffold materials rather than covering all regenerative strategies, extends the observation span to a complete 20-year timeline capturing the post-2020 boom of multifunctional biomaterials, and combines CiteSpace and VOSviewer for multi-dimensional triangulated visualization. Few prior similar analyses adopted this targeted material-centered framework, long-term time window and dual-software joint analysis simultaneously, enabling a more systematic and comprehensive depiction of the field’s evolutionary trajectory and emerging frontiers.

## Materials and methods

### Data collection

**Fig. 1:**
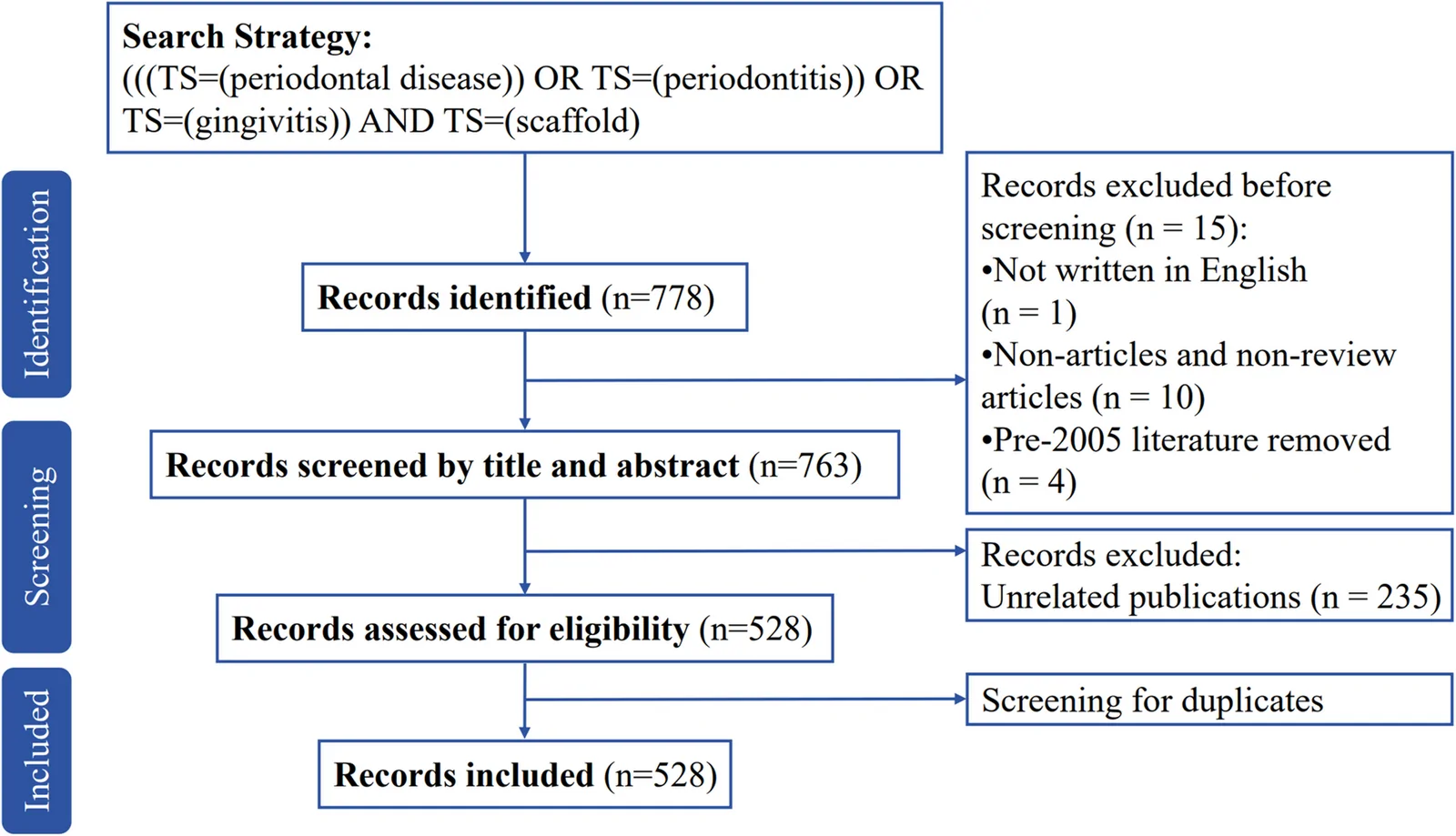
Flowchart of the literature search and selection process on the evolution of scaffold materials in periodontal disease. Records were identified from the Web of Science Core Collection (WoSCC) using a topic search that combined periodontal disease terms with scaffold-related terms (2005-2025). After deduplication and removal of ineligible records, two independent investigators screened titles, abstracts, and full texts. Numbers within boxes indicate the count of records at each stage

- Bibliometric data were collected from the Web of Science Core Collection (WoSCC), which served as a comprehensive database for scientific literature. Publications of interest were searched on July 30, 2025, using the following Boolean query: ((TS=(periodontal disease) OR TS=(periodontitis)) OR TS=(gingivitis)) AND TS=(scaffold). The search targeted studies investigating scaffold material applications in periodontal therapy, with publications from 2005 to 2025. The complete search strategy, including all Boolean operators and filters, is provided in the Supplementary Material.
- Screening criteria of initial records were set to include or exclude studies ***(***Fig. 1***)***. The inclusion criteria were review articles and peer-reviewed original research articles in English. The following exclusion criteria were applied: (1) literature not published in English; (2) document types other than original articles, such as meeting abstracts, editorials, letters, book chapters, and corrections; (3) literature published prior to 2005; (4) literature not related to scaffold materials for periodontal use; and (5) duplicate records of WoSCC sub-databases.

- Screening was done independently by two researchers. Title and abstract were screened first, followed by full-text assessment of qualified records. Disagreements were resolved by discussion or consulting a third investigator. Final included publications were exported as “Full Record and References Cited” for this study.
- The WoSCC database was selected as the sole data source because of its high indexing standards and reliable citation data, which are necessary for robust bibliometric network analysis using CiteSpace and VOSviewer. Although Scopus and Dimensions provide broader coverage, their metadata present specific limitations. Citation links are incomplete in Dimensions, while Scopus uses different citation matching criteria and requires additional preprocessing of citation records. These issues could compromise co-citation analysis. This decision may exclude some non-English or very recent articles, but the resulting bias is unlikely to materially affect the research findings.

### Data analysis

- Bibliometric analysis was carried out on the final corpus of 528 publications obtained from the systematic search. To analyze the evolution and intellectual structure of scaffold material research in periodontal clinical applications, CiteSpace (version 6.3.R3) was used, primarily for conducting bibliometric analysis. With its multifunctional capabilities, CiteSpace enabled visualization of citation and co-citation networks and thereby detecting landmark studies. It also facilitated the analysis of evolving keyword co-occurrence through timeline views and cluster analysis. This process specifically focused on research topic shifts and future research paradigm changes in the field of scaffold materials. CiteSpace parameters were set as follows: time slicing from 2005 to 2025 with a slice length of 1 year, selection criteria based on the g-index (k = 25), pruning was not applied, and all other options were kept at their default values.
- Comprehensive bibliometric map diagrams were produced using VOSviewer (version 1.6.20) with its default settings, including association strength normalization, modularity-based clustering, and the standard VOS layout. The graphs give prominence to co-authorship networks to present collaboration patterns between countries and institutions. Bibliographic coupling of sources was also represented by clustering journals according to shared references and co-occurrence networks from keywords in titles and abstracts. Collectively, the VOSviewer maps effectively represented the conceptual landscape, the thematic clusters, and the relative density and degree of interconnectedness of research themes pertaining to scaffold material types, applications, and outcomes in periodontology.

For country collaboration maps, a total link strength (TLS) threshold of > 4 was applied to retain countries with meaningful collaborative intensity and to reduce visual clutter caused by countries with only sporadic cooperation. The betweenness centrality threshold of > 0.1 was adopted from the CiteSpace default, which identifies nodes with a potentially important bridging function in the network.

## Results

### Temporal distribution of literature

**Fig. 2:**
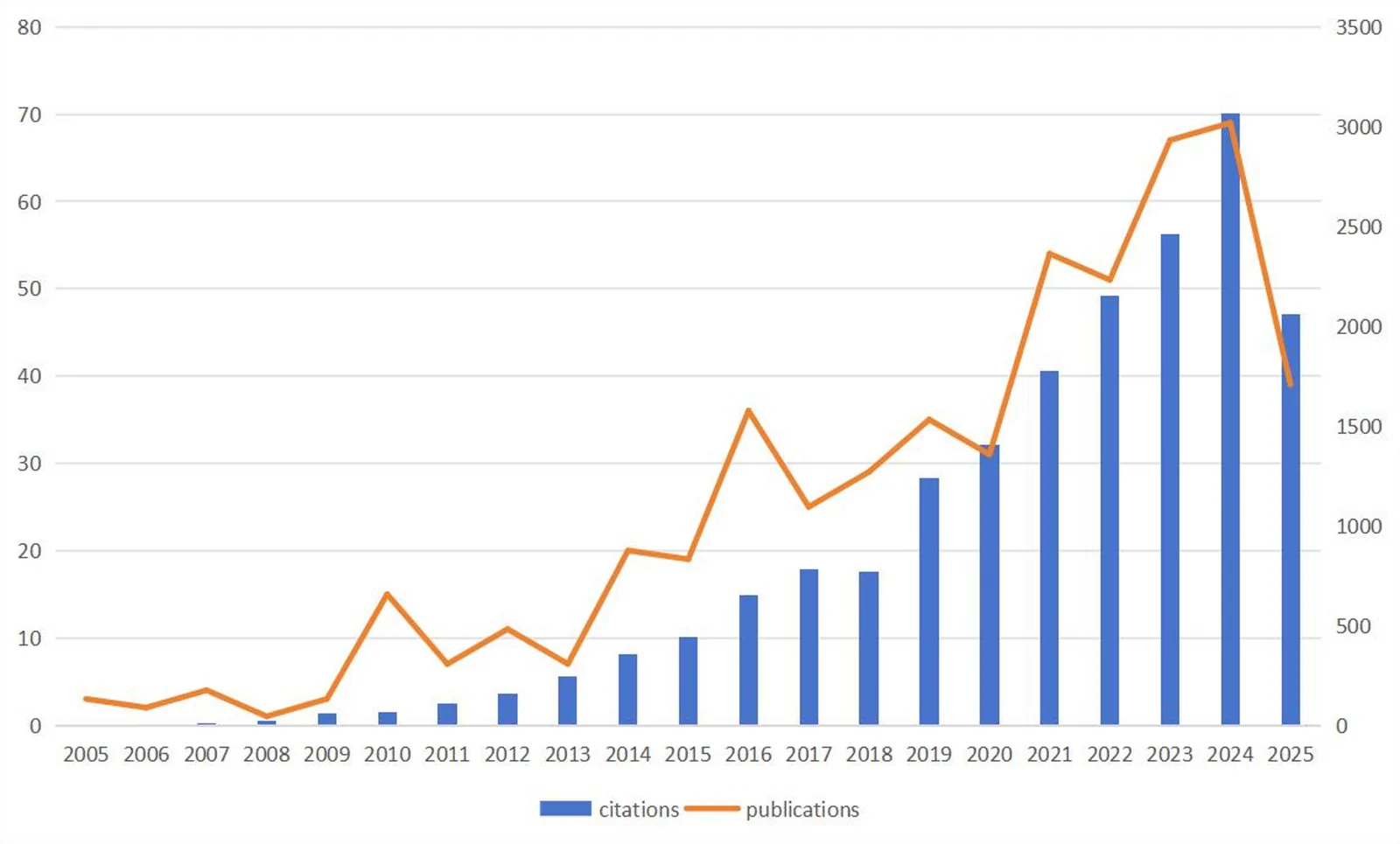
Trends in annual publications and citations for scaffold materials for periodontal therapeutics. Annual publication counts are shown as a line with symbols, and annual citation counts as bars

Figure 2 shows the two-decade trend in annual publication and citation. Numbers of publications and citations were flat across the range 2005–2008 and only exhibited fairly modest growth. However, in the interval since around 2015, cumulative publications and citations have continued to increase steadily with a clear spike following 2019. Above all, the latest five-year period (2020–2024) has been very productive with a remarkable contribution of over 60% to the total number of publications on which this current study is based. The greatest annual volume ever achieved was achieved by publications in 2024. Concurrently, citation numbers increased dramatically, which was also concentrated in the same recent five-year period (2020–2024), that accounted for more than 80% of the total citations received over the entire 20-year period. These statistics indicate a high increase in volume of research as well as author influence in the field of scaffold materials for periodontal applications in recent years. This post-2020 publication and citation boom signals the maturity of multifunctional biomaterial manufacturing techniques and rising clinical demand for translatable periodontal regenerative therapies, marking the transition from preliminary laboratory research to a high-priority interdisciplinary field integrating materials science and dentistry.

### Distribution of countries/regions

**Fig. 3:**
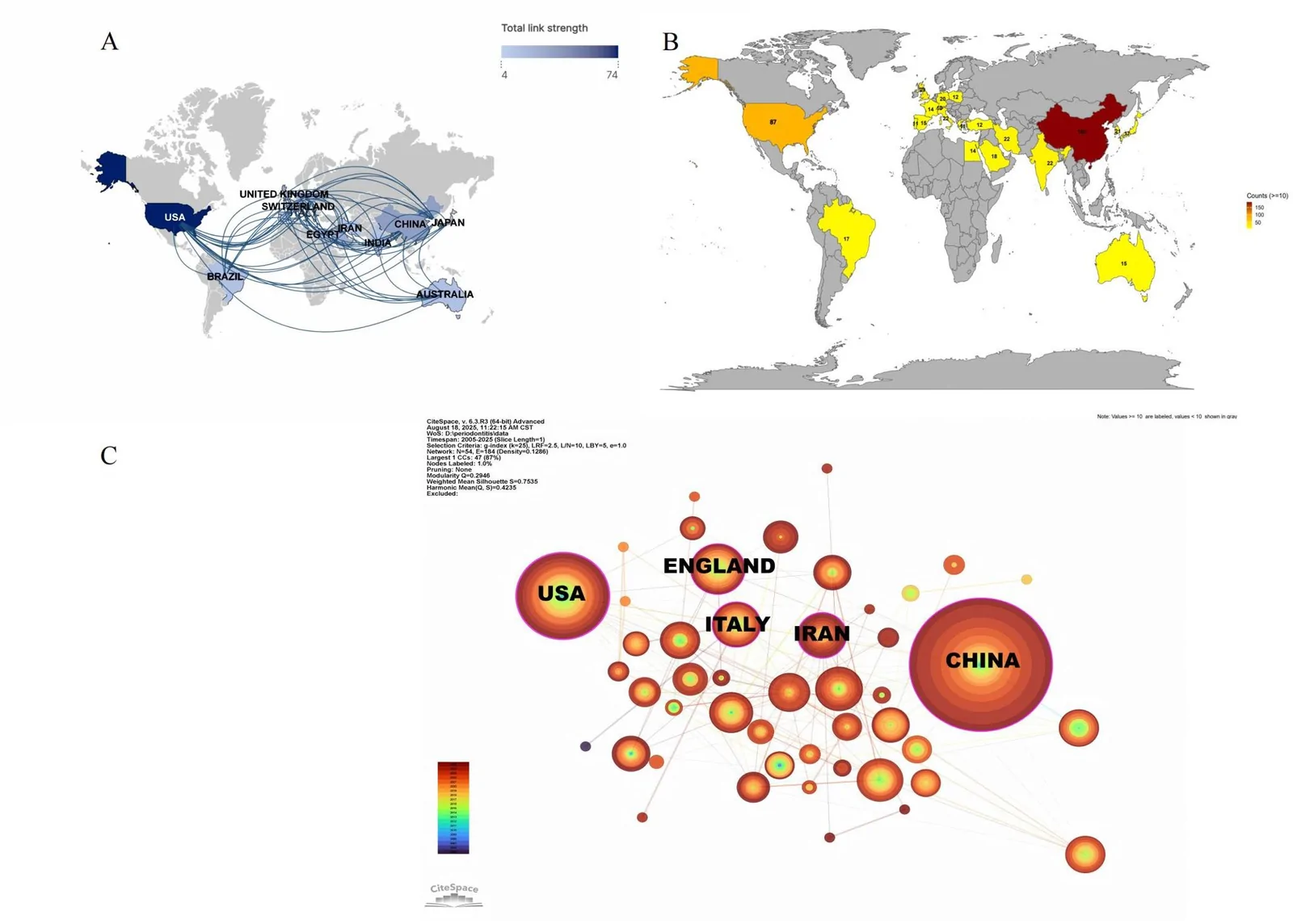
The global landscape of periodontal scaffold research across national contributions and collaborative networks. **A**. Geospatial mapping of international collaborative networks. Nodes represent countries/regions; lines represent co-authorship links. Color intensity indicates total link strength (TLS), with darker blue representing stronger collaboration. Countries with TLS below 4 are shown in gray. Node sizes are proportional to publication output. **B**. Global distribution of publication output by country/region. Country colors reflect publication counts, ranging from yellow for lower output to red for higher output. Countries with at least 10 publications are labeled, while those with fewer than 10 appear in gray. **C**. Network analysis of inter-country collaborative partnerships. Node size scales with publication volume, and link thickness reflects the strength of co-occurrence between countries. Node color indicates the average publication year, as shown by the color bar in the figure. Nodes with betweenness centrality greater than 0.1 are outlined in purple

The collaborative landscape in periodontal scaffold materials research is visualized through Fig. 3B. Geographic color intensity corresponds to national publication output while countries with publication counts above 10 are labeled and those below this threshold are shown in gray. China (185 publications) and United States (87 publications) emerge as core high-yield countries.

Then we analyze the international communication in the periodontal scaffold material field. The Fig. 3A presents a collaboration network diagram illustrating the strength of international partnerships, where the number of connecting lines indicates the intensity of cooperation between countries. Countries with a TLS value exceeding 4 are highlighted in blue, with darker shades indicating higher values. The US-Europe and China-Europe partnerships exhibit the strongest ties. Figure 3C displays the annual publication output of major countries engaged in research on this topic from 2005 to 2025 in the form of a co-occurrence network. It also shows each country’s yearly publication volume over this period (2005–2025). Nodes representing countries with a centrality value greater than 0.1 are highlighted with a purple outline in the diagram.

The above figures show that North America, Europe, and East Asia are the primary research centers globally, producing the most findings and serving as crucial connectors. This dual visualization strategy combines geographic density and network linkages, which effectively delineates global collaboration patterns driving advancements in periodontal therapeutics. Such concentrated geographic distribution stems from sufficient research funding and complete tissue engineering platforms in these three regions, and intensive cross-border collaborations facilitate rapid exchange of material fabrication and periodontal repair knowledge.

### Distribution of authors and research institutions

**Fig. 4:**
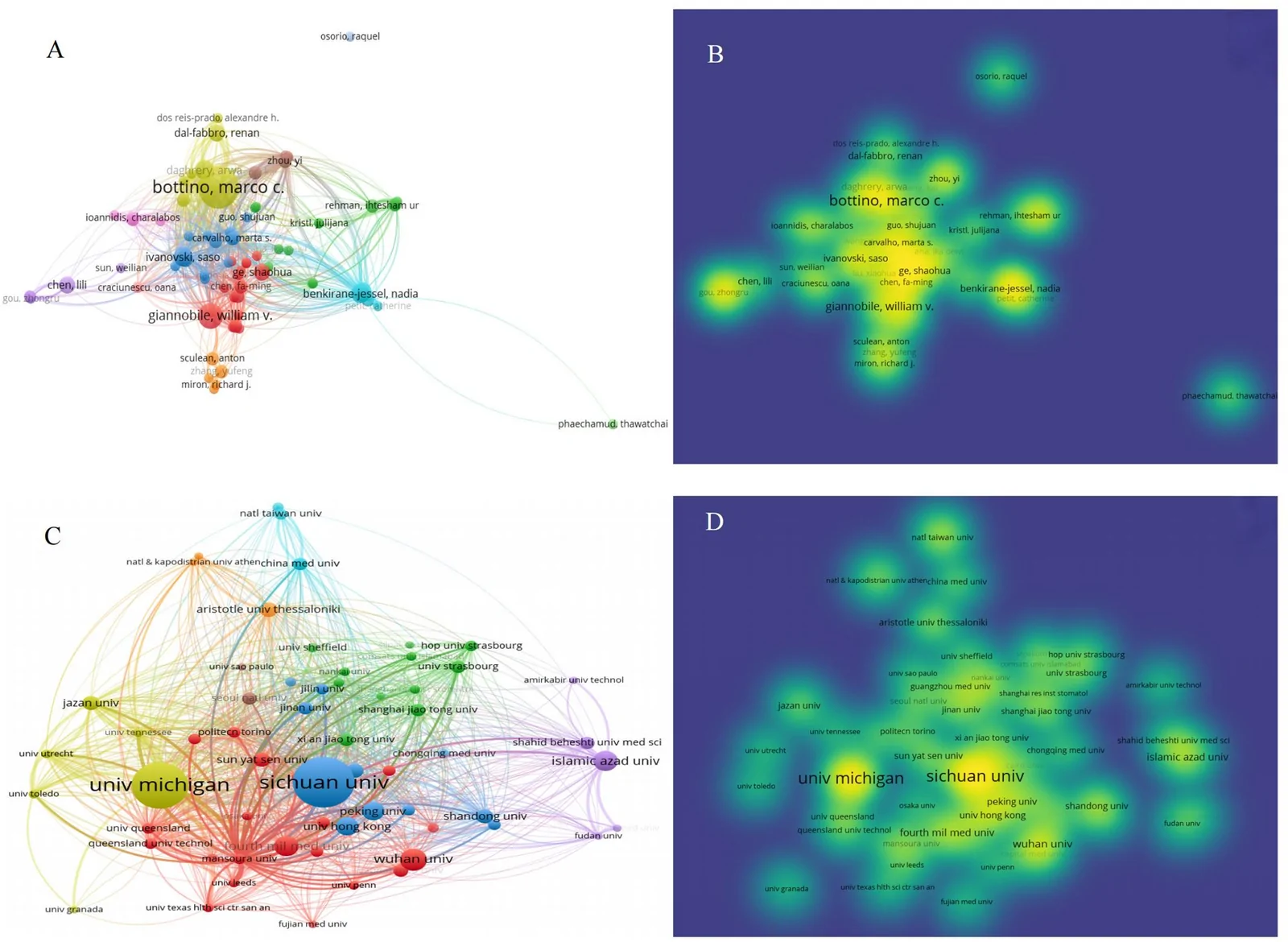
Collaborative network among authors and their institutional affiliations in periodontal scaffold research. **A**. Co-authorship network analysis among contributing researchers. Node size corresponds to the number of publications by each author. The number of connecting lines indicates how extensively an author collaborates, and node colors distinguish clusters of closely collaborating authors. **B**. Density visualization of the author co-authorship network. Brighter yellow areas represent higher density of collaboration. **C**. Inter-institutional collaborative network structure. Node size reflects the number of publications from each institution, and the number of connecting lines indicates the extent of collaboration. Node colors distinguish clusters of closely collaborating institutions. **D**. Density visualization of the institutional collaboration network. Brighter yellow areas represent higher density of collaboration

We used VOSviewer to map the co-authorship network among researchers in this field (Fig. 4A). In this network, node size represents the number of publications, and the number of connecting lines indicates the extent of collaboration. The most productive authors were ranked by publication count: Bottino, Marco C. topped the list with 14 articles, followed by Giannobile, William V. with 8 articles. Daghrery, Arwa, Ivanovski, Saso, Benkirane-Jessel, Nadia, and Huck, Olivier each contributed 6 articles. Among these authors, Bottino, Marco C. had the highest total citation count. The density of collaboration among authors is shown in Fig. 4B.

A total of 528 articles were identified in this research field. Fifteen institutions published more than 6 articles, representing 29.97% of the collected data. Table 1 lists the 15 most productive institutions ranked by publication count, including documents, citations, and TLS. Sichuan University, the University of Michigan, Wuhan University, and the Fourth Military University were the major contributors, publishing 26, 24, 11, and 10 articles, respectively, with total citation counts of 622, 1286, 267, and 1226. The collaborative network among these institutions is shown in Fig. 4C, and its density is visualized in Fig. 4D. The University of Michigan had the highest TLS of 7452, followed by Sichuan University with a TLS of 4241. Aggregation of productive authors and top-tier institutions indicates stable long-term academic teams dominate this field, which integrate cell biology, polymer materials and clinical periodontology to advance integrated scaffold-based regeneration strategies.

### Distribution of journals and co-cited references

In the analysis of journal distribution and co-cited references focusing on the evolution of scaffold materials for periodontal therapeutics using CiteSpace, journal quality was assessed using impact factor (IF) and Journal Citation Reports quartile rankings (Q1). The co-citation analysis identified leading cited journals in this specialized domain (Table 2), including Biomaterials (IF = 12.9, Q1), Journal of Periodontology (IF = 3.8, Q1), and Journal of Dental Research (IF = 5.9, Q1), which have consistently featured seminal works on scaffold-based periodontal regeneration. Pivotal co-cited references (Table 3), such as “Recent advances in periodontal regeneration: A biomaterial perspective” (2020) and studies on innovative injectable thermosensitive hydrogels (2019) have demonstrated high citation frequency and centrality, reflecting their foundational role in mapping the intellectual structure. Viewed together, these key publications reveal an ongoing combination of biomaterials, advanced stem cell technology, and precision fabrication, a combination that has shaped the evolution of scaffold strategies in periodontal therapy. The leading status of high-ranking biomaterial and professional dental journals proves that innovative periodontal scaffold materials require dual validation of material mechanism research and clinical therapeutic efficacy, establishing solid intellectual foundations for subsequent translational studies.

### Disciplinary distribution and journal mapping

**Fig. 5:**
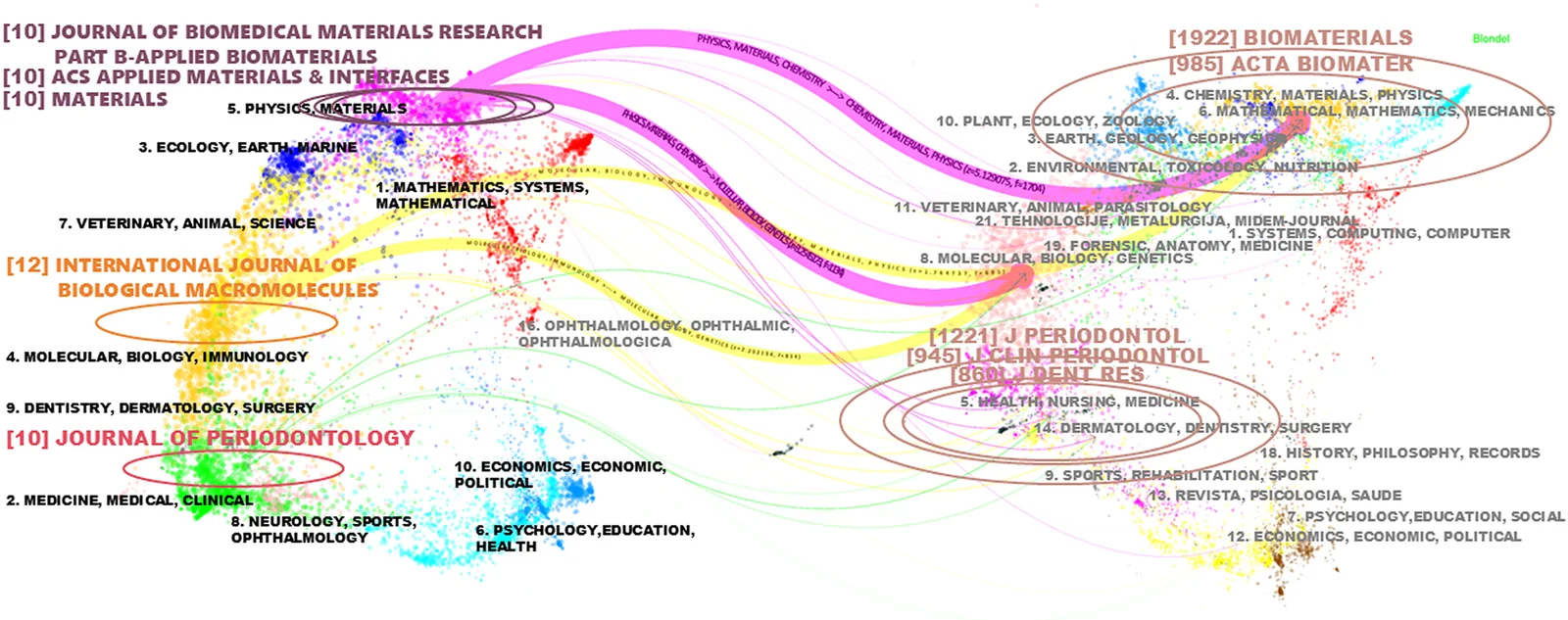
Dual map overlay of journals on periodontal scaffold materials. In this overlay, nodes on the left represent citing journals, and nodes on the right represent cited journals. Node colors mark disciplinary categories. Colored curves trace citation flows from left to right, with curve color matching the disciplinary category of the citing journal. Thicker curves indicate stronger citation relationships

A dual map overlay diagram shown in Fig. 5 is about the relationship between the citing and cited journal and the disciplines within. The journals on the left cite the ones on the right. as references. Different colors were used for different journal articles. The thickness of path between the citing and cited journals represents their association strength. There are two prominent links, both with citing disciplines of physics, materials and chemistry. The thickest link is most cited by journals within its same disciplines, physics, materials and chemistry (f = 1704). The second highest frequency shows that the research in molecular biology and genetics is most cited by journals about physics, materials and chemistry (f = 1134). This cross-disciplinary citation pattern demonstrates that periodontal scaffold material research is material-oriented; material chemistry and manufacturing serve as core knowledge basis, while molecular biology provides theoretical support for functional modification of scaffold materials.

### Classification of scaffold materials

Table 4 shows the top 20 keywords, which are listed in a descending order of count number. Centrality is an indicator for the importance of keywords, and 0.1 is the threshold value. Based on the bibliometric analysis, the research landscape was initiated by foundational in vitro studies (2005) exhibiting the highest network centrality (0.11), which progressively transitioned toward scaffold-based research (*n* = 139) with the centrality of 0.08 peaking in 2009 alongside emerging stem cell differentiation research like osteogenic differentiation (2007) with the centrality of 0.09. A significant thematic shift occurred after 2011 with more focus on clinical research, evidenced by increasing frequency of bone regeneration (2011) and delivery systems (2014). The persistent prominence of scaffold materials and the sustained bridging role of in vitro methodologies highlight enduring priorities in the field.

**Fig. 6:**
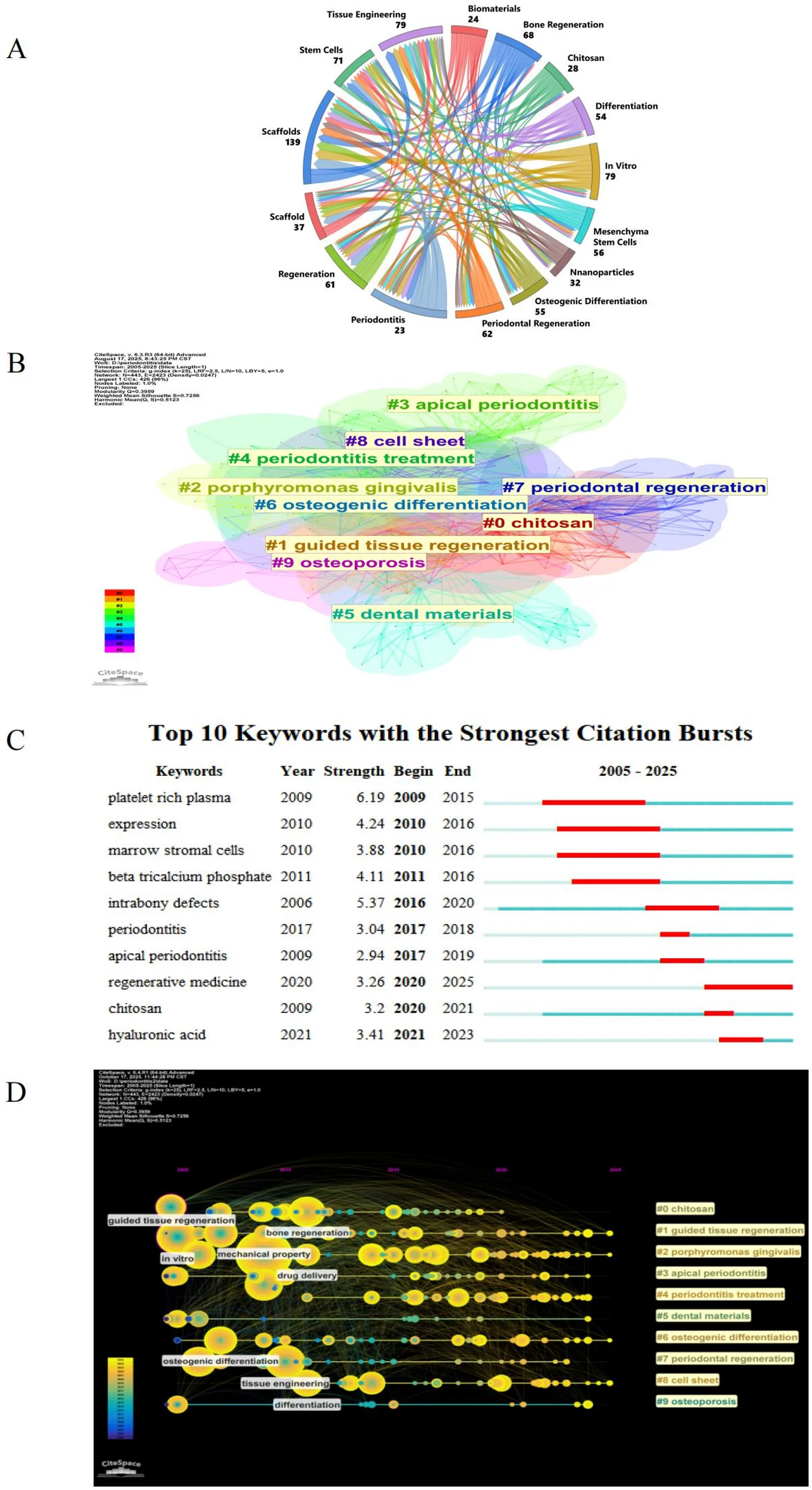
Co-occurrence analysis of keywords related to scaffold materials in periodontal therapeutics. **A**. Chord diagram of keyword co-occurrence relationships. Arc width reflects the occurrence frequency of each keyword, and chord thickness indicates the strength of co-occurrence between keywords. Keyword labels and their occurrence frequencies are shown in the figure. **B**. Co-occurrence frequency of the top 10 keywords within clusters. Colors represent different keyword clusters, and cluster labels (#0-#9) are indicated in the figure. **C**. Top 10 keywords with the strongest citation bursts. Each keyword is shown as a horizontal bar. Blue segments indicate the overall time span, while red segments mark the period of citation burst. Burst strength is listed next to each keyword, and keywords are ordered by the start year of the burst. **D**. Timeline view of the temporal evolution of keyword clusters. Nodes represent keywords, with size proportional to keyword frequency. Node color indicates the year of publication, shown as concentric rings. Lines between nodes represent co-occurrence relationships, and the number of lines reflects association strength. Nodes with betweenness centrality greater than 0.1 are outlined in purple

In Fig. 6A, a chord diagram of the keywords “co-occurrences” is displayed. The association strength between them represented by the thickness of path. There are 15 keywords selected in total, which are listed in descending order of occurrences: scaffolds (*n* = 172), periodontitis (*n* = 123), regeneration (*n* = 85), tissue engineering (*n* = 82), in-vitro (*n* = 80), scaffold (*n* = 72), bone regeneration (*n* = 68), periodontal regeneration (*n* = 67), stem-cells (*n* = 62), differentiation (*n* = 58), osteogenic differentiation (*n* = 57), mesenchymal stem-cells (*n* = 55), biomaterials (*n* = 48), chitosan (*n* = 46) and nanoparticles (*n* = 40). In terms of their link strength, “scaffolds” is most related to other keywords with a total of 322 linked articles. Also notably, scaffolds usually occurred following with other keywords denoting by many arrows pointing into it.

Figure 6B identifies a total of 10 cluster of keywords, which were: #0 chitosan, #1 guided tissue regeneration, #2 Porphyromonas gingivalis, #3 apical periodontitis, #4 periodontitis treatment, #5 dental materials, #6 osteogenic differentiation, #7 periodontal regeneration, #8 cell sheet and #9 osteoporosis.

The burst analysis in Fig. 6C identifies distinct research trends, initially dominated by biological factors like platelet rich plasma with the highest citation burst value of 6.19 between 2009 and 2015 and cellular investigations like marrow stromal cells with the burst value of 3.88 between 2010 and 2016. Then, it was subsequently transitioned to biomaterial research like beta tricalcium phosphate (citation burst = 4.11 between 2011 and 2016) and targeted clinical applications, such as intrabony defects (citation burst = 5.37 between 2016 and 2020), periodontitis (citation burst = 3.04 between 2016 and 2020) and apical periodontitis (citation burst = 2.94 between 2017 and 2019). Recently, there was a surge in regenerative medicine like ongoing (citation burst = 3.26 since 2020) and polymers material such as chitosan (citation burst = 3.2 between 2020 and 2021) and hyaluronic acid (citation burst = 3.41 between 2021 and 2023). The burst diagram reveals a clear evolution from foundational biological investigation toward clinical research.

### Keyword clustering

The clustering of title-derived keywords resulted in ten thematic clusters. Figure 6D shows how these keywords have appeared over time. The clustering gave us a Q (modularity) of 0.3959 and a weighted average silhouette S of 0.7259, which suggest the network is well structured and the clusters are reliable. Among the cluster labels, there were “guided tissue regeneration”, “bone regeneration”, “mechanical property”, “drug delivery”, “in vitro”. The chronological shift of core keywords, citation burst hotspots and thematic clusters fully reflects the developmental logic of this field: from basic in vitro cell experiments to clinical bone repair strategies, and eventually multifunctional biomaterial scaffold materials designed for clinical periodontal defect treatment, revealing a complete lab-to-clinic translational trajectory.

## Discussion

Periodontitis is a chronic inflammatory condition driven by many causes. It starts with plaque buildup and slowly eats away at the tissues that hold teeth in place: the periodontal ligament and alveolar bone.(Slots 2017). Due to the lack of early symptoms, periodontitis often goes unnoticed until it becomes severe enough to cause tooth loss, typically with gingivitis, clinical attachment loss, radiographic evidence of bone loss, etc. (Brunsvold 2005; Page and Eke 2007; Papapanou et al. 2018). In response to this clinical challenge, tissue engineering regeneration strategies demonstrate considerable potential. To rebuild the periodontal tissue, different scaffold materials have been applied to rebuild the periodontal tissue(Woo et al. 2021). This study analyzed all 528 articles obtained from the systematic search described in Sect.  2.1 to trace the development trajectory and knowledge structure of scaffold materials in periodontal clinical applications, indicating that periodontal scaffold material research has evolved into a dynamic field of study and are accelerating towards functionalisation and clinical application.

With the transition from gradual to rapid growth, research have shifted from in vitro basic experiments to clinically-oriented studies(Apatzidou et al. 2021). The earliest research began with in vitro models(Hasegawa et al. 2005; Pang et al. 2005), the differentiation process of stem cells(Baba et al. 2016; Chen et al. 2012, 2016; Kawaguchi et al. 2004), and has evolved towards clinically relevant topics, including bone regeneration (Goudouri et al. 2016; Luo et al. 2024; Singh et al. 2015) and targeted drug delivery(Budală et al. 2023; Ferreira et al. 2021; Lima de Sousa et al. 2024; Song et al. 2022). The rapid growth in the number of current publications and citation rates, especially from 2020 to 2024, which account for over 60% of the total and over 80% of total citations, indicates an academic explosion (Fig. 2). This not only reflects changes in research interests but also the demands of clinical treatment. Overall, both clinical and experimental research systematically show that this field has entered a phase of integration and rapid expansion, characterised by deeper integration of basic biology, material functionalisation and clinical requirements.

The global distribution of literature is highly concentrated. It mainly manifested in the formation of a global research network with the America and China, the two countries with the highest output of literature, and Europe. In terms of output institutions (Sect.  3.3), the top three institutions in terms of publication volume are Sichuan University (26 articles) and the University of Michigan (24 articles), and they have high network connectivity (with TLS values of 7452), becoming key nodes in international cooperation. Such types of collaborations have significantly advanced the cross-border flow of resources, technologies, and ideas, such as the development of new scaffold materials like three-dimensional (3D)-printed scaffold materials(Theodoridis et al. 2023; Wang et al. 2021) and nanocomposite hydrogels(Dos Santos et al. 2024; Liu et al. 2024; Zhu et al. 2025), demonstrating that pioneering innovation increasingly relies on the global integration of resources and intellectual capital.

More importantly, the frontiers of research on new scaffold materials and their translation focus on functionalized scaffold materials, mainly in hydrogels(Pan et al. 2025; Ran et al. 2024), nanocomposites(Nasiri et al. 2023; Wang et al. 2024), and 3D-printed structures(Lee et al. 2014; Sufaru et al. 2022). Current research focuses on introducing immunomodulatory and antibacterial functions to address the complex needs of periodontal tissue regeneration. For example, a 2019 study on a thermosensitive hydrogel as a kind of scaffold material that controlled the release of aspirin and erythropoietin demonstrated the effects of periodontal tissue regeneration and anti-inflammation(Xu et al. 2019). Moreover, polymer materials such as chitosan(Geng et al. 2023; Shariatinia 2018; Zhang et al. 2025) and hyaluronic acid(Olszewska-Czyz et al. 2021; Santos et al. 2023; Sukumar and Drízhal 2007) have shown explosive growth since 2020, indicating that scaffold material design emphasizes fundamental issues such as balanced biocompatibility and functionality. In addition, a recent randomized controlled trial study published by Wang et al. demonstrated the excellent efficacy and optimal wound healing effect of the modified collagen scaffold (ribosomally cross-linked collagen membrane, RCCM)(Yiwei Wang et al. 2025a, b).

The dynamic evolution of keyword co-occurrence and topic clustering reveals the logical relationship of research themes. The keywords “scaffolds” (172), “periodontitis” (123), and “regeneration” (85) jointly highlight a continuous theme - “materials-disease-effectiveness”, which represents the logical process of research. Based on burst analysis, regenerative medicine will continue to be the dominant research paradigm, while functionalised polymeric materials such as hyaluronic acid represent the next frontier in scaffold material development through key techniques such as 3D printing. In the clustering, the distinction of chitosan, guided tissue regeneration, and periodontal regeneration themes reflects the intersection of material chemistry and clinical application. According to the burst analysis results, the shift in research focus can be captured, such as the appearance of keywords related to bone defects and periodontitis after 2016, indicating a shift from basic mechanisms to clinical treatment. However, this research trend highlights a core challenge that clinical trials are necessary to properly assess the therapeutic effects of these important technologies(Hu et al. 2025). Currently, certain periodontal scaffold materials, such as collagen scaffolds(Solderer et al. 2024), have entered the mid-to-late stages of clinical translation and are evolving toward functionalization by incorporating substances like Platelet-Rich Fibrin (NCT07081230), growth factor (CTRI/2024/09/073701), and human fibroblast growth factor (CTRI/2025/08/093282, CTRI/2025/07/091569). However, multifunctional emerging materials—including thermosensitive hydrogels and 3D-printed scaffolds—remain in the basic research phase within periodontal therapy and have yet to enter clinical studies.

Looking ahead, periodontal therapeutic scaffold material research is expected to move toward data-driven and patient-specific regenerative platforms. AI-assisted biofabrication may enable the optimization of scaffold material architecture, bioink composition, and manufacturing parameters, while image-guided design and personalized 3D printing could generate multiphasic, anisotropic constructs that more closely reproduce the cementum-periodontal ligament–alveolar bone interface(Dai et al. 2025). The clinical translation of these systems will depend on reproducible vascularization, controlled immunomodulation, long-term mechanical stability, scalable manufacturing, standardized workflows, and clearly defined regulatory and safety pathways, supported by well-designed clinical studies(Daghrery et al. 2026). In parallel, integration of genomics, transcriptomics, proteomics, metabolomics, and microbiome data with bioinformatics and machine-learning models may identify patient- and microenvironment-specific regenerative targets, thereby supporting precision scaffold material selection and design(Jin et al. 2025). Smart responsive biomaterials that sense pH, enzymes, inflammatory mediators, or other local cues and provide spatiotemporally controlled antimicrobial, immunomodulatory, and osteogenic functions represent a further direction toward adaptive periodontal therapy(Li et al. 2025). Collectively, the field is likely to progress from passive defect-filling scaffolds toward integrated, digitally designed, biologically instructive, and clinically deployable regenerative systems.

## Limitations of study

There exist several limitations to this research, primarily as a result of the literature filtering criteria. Omitting a category of publication, such as conference proceedings, technical reports, editorials, and letters to the editor, might result in the new trends and valuable expert opinions not having been captured. Restricting the search to documents published after 2005 might omit older fundamental studies offering the background and historical context to the discipline. Moreover, the removal of duplicates across databases could inadvertently exclude relevant studies indexed in more than one database. The study solely utilizes the WoS database and could overlook influential studies from non-covered databases. It should be noted that PubMed, despite being a major biomedical database, was not used because it does not provide the structured, exportable data fields required for co-authorship networks, institutional collaboration analyses, and co-citation analysis in tools such as CiteSpace and VOSviewer. Perhaps the addition of patent and clinical database searching would be more effective. Furthermore, older papers naturally accumulate more citations due to longer exposure, which can possibly distort the visibility of more recent yet seminal work. Certain inherent drawbacks of bibliometric techniques like citation biases and the constantly evolving nature of scholarly fashion also remain on the loose and are difficult to counter.

Notably, citation bias represents an inherent flaw of bibliometric evaluation. Older publications naturally accumulate more citations over extended exposure time, which disproportionately inflates their apparent impact and centrality within co-citation and keyword burst networks. This temporal citation accumulation effect obscures the actual academic value of recently published pioneering studies on 3D-printed scaffolds, immunomodulatory hydrogels and nanocomposites, as these cutting-edge materials lack sufficient time to gather adequate citation counts. Therefore, pure citation-based bibliometric indicators cannot fully and objectively reflect the real innovative potential of recent breakthroughs in periodontal scaffold material research.

Crucially, all bibliometric indicators adopted in this analysis, including publication volume, citation frequency, node centrality, and keyword burst strength, are purely quantitative statistical outputs. These metrics fail to quantitatively or qualitatively assess the methodological quality of individual publications, such as experimental design rationality, sample representativeness, control group setting, reproducibility of in vitro/in vivo protocols, and risk of bias in clinical trials. Additionally, citation-based bibliometric evidence cannot independently judge the actual clinical transformative value or patient-oriented therapeutic benefits of a single piece of research. High-cited articles may contain flawed experimental schemes, while low-cited recent works with rigorous methodology and promising clinical prospects could be underestimated, which means bibliometric results should only be interpreted together with manual content evaluation rather than as a sole standard to judge research merit.

## Conclusions

This bibliometric analysis delivers practical insights for Saudi Dental Journal (SDJ) audiences. Basic researchers studying scaffold materials for periodontal therapeutics can identify core cooperation networks and emerging hotspots of periodontal regeneration to guide research planning. Clinical periodontists gain a clear overview of multifunctional biomaterial translation gaps, helping them understand the prospects of regenerative therapies for routine periodontal therapeutics. This interdisciplinary overview matches SDJ’s academic and clinical readership.

Over the past 20 years, we have seen real progress in the research of scaffold materials in periodontal treatment, shifting from basic in vitro studies to clinically-oriented regenerative methods. In recent years, the significant increase in related publications and citations indicates growing interest in this field among both the academic community and the practice domain. Initially, research focused on basic areas such as tissue engineering and stem cell differentiation, but gradually shifted to application areas such as bone regeneration, targeted drug delivery, and the development of advanced biomaterials. International cooperation among leading research countries has played a crucial role in promoting innovation and interdisciplinary knowledge exchange.

The current emerging trend is centered on the design of functional scaffolds, such as hydrogels, nanocomposites, and 3D-printed structures, which have enhanced immune modulation and antibacterial properties. Keyword analysis further indicates that the research topic has shifted from basic concepts to clinically relevant and material-driven research directions. Although early research mainly focused on in vitro models and mechanistic insights into stem cell behavior, recent efforts have increasingly pointed towards translational applications involving new materials and personalized treatment strategies. These advancements lay the foundation for the development of multifunctional, patient-specific scaffolds and comprehensive regenerative treatments, potentially improving the clinical efficacy of periodontal treatment.

Collectively, this two-decade bibliometric mapping confirms that the combined advancement of multifunctional, immunomodulatory scaffold materials and long-term cross-border collaborative networks constitutes the irreplaceable core engine for translational periodontal regeneration; sustained international knowledge exchange and interdisciplinary material innovation will continuously bridge laboratory material fabrication and standardized clinical periodontal therapeutics, unlocking predictable, personalized regenerative treatment outcomes for patients with severe periodontitis.

**Table 1:** Top 15 institutions with the most publications on scaffold materials in periodontal therapeutics

Rank	Organization	Documents	Citations	Total link strength
1	Sichuan University	26	622	4241
2	University of Michigan	24	1286	7452
3	Wuhan University	11	267	807
4	Fourth Military Medical University	10	1226	3603
5	Islamic Azad University	10	236	2010
6	Peking University	9	347	1863
7	The University of Hong Kong	9	208	1852
8	Aristotle University of Thessaloniki	8	68	1196
9	Jazan University	7	156	2596
10	Sun Yat-sen University	7	126	1605
11	Shandong University	7	188	1299
12	Zhejiang University	7	83	745
13	University of Strasbourg	6	385	1496
14	Xi’an Jiaotong University	6	336	1399
15	Shahid Beheshti University of Medical Sciences	6	234	1298

Abbreviations: TLS, total link strength. Total link strength reflects the overall strength of an institution’s co-authorship links with all other institutions; higher values indicate stronger collaborative intensity

**Table 2:** Top 15 cited journals in publications on scaffold materials for periodontal therapeutics

Rank	Cited Journals	IF	JCR	Frequency
1	BIOMATERIALS	12.9	Q1	376
2	J PERIODONTOL	3.8	Q1	294
3	J DENT RES	5.9	Q1	281
4	J CLIN PERIODONTOL	6.8	Q1	277
5	ACTA BIOMATER	9.6	Q1	236
6	J BIOMED MATER RES A	3.9	Q1	231
7	PERIODONTOL 2000	15.7	Q1	230
8	MAT SCI ENG C-MATER	/	Q1	213
9	J PERIODONTAL RES	3.4	Q1	203
10	TISSUE ENG PT A	2.9	Q1	192
11	INT J MOL SCI	5.7	Q1	175
12	SCI REP-UK	4.3	Q1	163
13	DENT MATER	6.3	Q1	160
14	J BIOMED MATER RES B	4.4	Q1	158
15	PLOS ONE	3.2	Q1	155

Abbreviations: IF, Impact Factor; JCR, Journal Citation Reports. Frequency refers to the number of times a journal was cited in the included publications

**Table 3:** Top 15 cited references on scaffold-based periodontal therapeutics

Rank	Frequency	Title	Journal	Author	Year
1	30	Recent advances in periodontal regeneration: A biomaterial perspective	BIOACT MATER	Liang YX	2020
2	23	An injectable and thermosensitive hydrogel: Promoting periodontal regeneration by controlled-release of aspirin and erythropoietin	ACTA BIOMATER	Xu XW	2019
3	20	The recent advances in scaffolds for integrated periodontal regeneration	BI0ACT MATER	Woo HN	2021
4	17	Tri-Layered Nanocomposite Hydrogel Scaffold for the Concurrent Regeneration of Cementum, Periodontal Ligament, and Alveolar Bone	ADV HEALTHC MATER	Sowmya S	2017
5	16	Periodontal diseases	NAT REV DIS PRIMERS	Kinane DF	2017
6	16	Periodontal Bone-Ligament-Cementum Regeneration via Scaffolds and Stem Cells	CELLS-BASEL	Liu J	2019
7	15	Multiphasic scaffolds for periodontal tissue engineering	J DENT RES	Ivanovski S	2014
8	15	Advance of Nano-Composite Electrospun Fibers in Periodontal Regeneration	FRONT CHEM	Zhuang Y	2019
9	15	Periodontal Tissue Engineering with a Multiphasic Construct and Cell Sheets	J DENT RES	Vaquette C	2019
10	14	Treatment of periodontal intrabony defects using autologous periodontal ligament stem cells: a randomized clinical trial	STEM CELL RES THER	Chen FM	2016
11	13	Highly tunable bioactive fiber-reinforced hydrogel for guided bone regeneration	ACTA BIOMATER	Dubey N	2020
12	13	Sustained Release of Two Bioactive Factors from Supramolecular Hydrogel Promotes Periodontal Bone Regeneration	ACS NANO	Tan JL	2019
13	12	New biodegradable nanoparticles-in-nanofibers based membranes for guided periodontal tissue and bone regeneration with enhanced antibacterial activity	J ADV RES	Abdelaziz D	2021
14	11	Regenerative Medicine for Periodontal and Peri-implant Diseases	J DENT RES	Larsson L	2016
15	11	Tissue engineering bone-ligament complexes using fiber-guiding scaffolds	BIOMATERIALS	Park CH	2012

Abbreviations: Frequency refers to the number of times each reference was cited in the included publications

**Table 4:** Top 20 keywords in scaffold materials for applying in periodontal diseases

Rank	Count	Centrality	Year	Keywords
1	139	0.08	2009	scaffolds
2	79	0.11	2005	In vitro
3	79	0.10	2010	Tissue engineering
4	71	0.06	2009	Stem cells
5	68	0.07	2011	Bone regeneration
6	62	0.07	2006	Periodontal regeneration
7	61	0.03	2006	regeneration
8	56	0.07	2007	Mesenchymal stem cells
9	55	0.08	2007	Osteogenic differentiation
10	54	0.09	2011	differentiation
11	43	0.06	2014	delivery
12	40	0.06	2007	Tissue regeneration
13	39	0.05	2009	bone
14	37	0.01	2017	scaffold
15	37	0.08	2005	tissue
16	35	0.01	2005	Guided tissue regeneration
17	32	0.02	2014	nanoparticles
18	31	0.06	2011	Drug delivery
19	28	0.04	2009	chitosan
20	25	0.03	2018	membranes

Abbreviations: Centrality refers to betweenness centrality in the keyword co-occurrence network; higher values indicate a stronger bridging role

## Supplementary Information

Below is the link to the electronic supplementary material.

Supplementary Material 1

## Data Availability

The bibliometric data used in this study were retrieved from the Web of Science Core Collection (WoSCC), a subscription-based database. The complete search strategy and Boolean query are provided in Supplementary File. The exported full record dataset is available from the corresponding author upon reasonable request.
