# Supplementary material for: Evolution of scaffold materials for periodontal therapeutics over two decades (2005–2025): A bibliometric mapping analysis
Source: Saudi Dent J. 2026 Sep 21;38(10):125. doi: 10.1007/s44445-026-00235-y (PMC13594021; doi:10.1007/s44445-026-00235-y)
Supplement: Supplementary file 1 [file 44445_2026_235_MOESM1_ESM.pdf]

Complete search strategy for Web of Science Core Collection (WoSCC)

Database: Web of Science Core Collection (WoSCC)
Search date: July 30, 2025
Search query:
((TS=(periodontal disease) OR TS=(periodontitis)) OR TS=(gingivitis)) AND TS=(scaffold)
After retrieval, the following filters were applied in the Web of Science interface:
Time span: 2005-01-01 to 2025-07-30
Language: English
Document type: Article or Review Article
These filters excluded 15 records from the initial 778 retrieved records: 1 non-English record, 10 non-article/review records, and 4 records published before 2005. A total of 763 records remained for title/abstract screening, as shown in Figure 1.
